# Orders of Magnitude Extension of the Effective Dynamic Range of TDC-Based TOFMS Data Through Maximum Likelihood Estimation

**DOI:** 10.1007/s13361-014-0961-5

**Published:** 2014-07-22

**Authors:** Andreas Ipsen, Timothy M. D. Ebbels

**Affiliations:** 1 Institute of Mass Spectrometry, College of Medicine, Swansea University, Swansea, SA2 8PP UK; 2 Computational and Systems Medicine, Department of Surgery and Cancer, Faculty of Medicine, Imperial College London, London, SW7 2AZ UK

**Keywords:** Time-of-flight mass spectrometry, Maximum likelihood estimation, Dynamic range, Time to-digital converter, Statistics, Dead time, Detector saturation

## Abstract

In a recent article, we derived a probability distribution that was shown to closely approximate that of the data produced by liquid chromatography time-of-flight mass spectrometry (LC/TOFMS) instruments employing time-to-digital converters (TDCs) as part of their detection system. The approach of formulating detailed and highly accurate mathematical models of LC/MS data via probability distributions that are parameterized by quantities of analytical interest does not appear to have been fully explored before. However, we believe it could lead to a statistically rigorous framework for addressing many of the data analytical problems that arise in LC/MS studies. In this article, we present new procedures for correcting for TDC saturation using such an approach and demonstrate that there is potential for significant improvements in the effective dynamic range of TDC-based mass spectrometers, which could make them much more competitive with the alternative analog-to-digital converters (ADCs). The degree of improvement depends on our ability to generate mass and chromatographic peaks that conform to known mathematical functions and our ability to accurately describe the state of the detector dead time—tasks that may be best addressed through engineering efforts.

**Electronic supplementary material:**

The online version of this article (doi:10.1007/s13361-014-0961-5) contains supplementary material, which is available to authorized users.

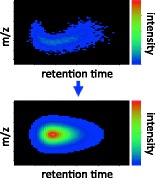

## Introduction

The analysis of LC/MS data requires solutions to a number of non-trivial data analytical problems and, by now, a very wide range of heuristic algorithms have been developed to address them [1–5]. But interestingly, well-established tools from frequentist statistics such as hypothesis testing and maximum likelihood estimation are generally not used in this context. We believe that this is in large part because the probability distributions governing the data-generation processes of instruments as elaborate as high-throughput mass spectrometers have been assumed to be too complicated to allow for a manageable mathematical formulation. But as we demonstrated in a recent article [6], it is in fact possible to describe the distribution of the raw data produced by time-of-flight mass spectrometers employing time-to-digital converters, through a relatively simple binomial-type model, which we refer to as the “basic model”.

We stress that this model is different in character from most alternatives because it has been derived from an understanding of the data-generation process and not simply designed to “resemble” empirical data by its qualitative features. It is also capable of describing real TOFMS data far more accurately than any other model we are aware of, and its predictions have been validated in a manner that is far more statistically rigorous than any alternatives we have been able to find. Moreover, through its parameters and their joint relationships it can formally relate features of analytical interest, such as the mass or isotope pattern of an analyte or whether two coeluting compounds are products of the same precursor, to the distribution of experimental data.

One of the major virtues of devising such probability distributions to describe acquired data is that they allow for very precise formulations of common data analytical problems as well as procedures for obtaining “optimal” solutions to them. For example, the problem of TDC saturation, which will be addressed here, will be framed as an estimation problem in which the true rate of ion arrivals is a parameter in the basic model whose value is estimated by means of the method of maximum likelihood. Maximum likelihood estimators (MLEs) are amongst the most established tools of statistical theory and, in addition to their intuitive appeal, they have highly desirable properties in the limit of large sample sizes [7]. Their proper use in mass spectrometry—which requires that the probability distribution of the acquired data is known—should arguably be a central aim in the development of MS data analytical methods.

TDCs are generally cheaper than the alternative ADCs, and also have finer time resolution, and are resistant to electronic noise and to the variable detector gain. Nevertheless, many TOFMS manufacturers have been moving away from TDCs, instead adopting ADCs due to the latter’s wider dynamic range. Although statistical corrections that improve the dynamic range of TDC-based instruments have long been available, they have hitherto not been powerful enough to render such instruments competitive with ADC-based alternatives. But as will be shown below, this might well be possible if greater efforts were made by instrumentalists to closely tailor their instruments to the requirements of the statistical analysis of the output data.

## Theory

If we observe a compound over *N* distinct chromatographic scans and *M* distinct *m/z* bins, we consider the distribution of the *N* × *M* observed ion counts, which can be labeled *k*_*1,1*_,…, *k*_*N,M*_. The basic model provides the probability, P(*k*_*1,1*_,…, *k*_*N,M*_ | *θ*_Ω_, *θ*_*Γ*_, *I*) (Equation 5 of the Supplementary Information) of observing these counts, given the parameters of the mass and chromatographic peaks, which will be labeled *θ*_Ω_ and *θ*_Γ_, respectively, as well as the average number of ion arrivals over the entire peak, *I*.

The basic model corrects for saturation by finding the value of *I* that has the highest possible probability of inducing the *k*_*1,1*_,…, *k*_*N,M*_ observed in the data. This correction is most powerful if the functional forms of both the mass and chromatographic peaks are known, in which case the correction is applied jointly to the full set of ion counts—a procedure that will be labeled correction method (1). Alternatively, if we know the shape of the mass peak but not of the chromatographic peak, we may apply the correction separately to estimate the average number of ion arrivals of the mass peaks observed in each distinct chromatographic scan [correction method (2)]. And finally, if neither peak shape is known, correction method (3) estimates the average number of ion arrivals in each of the *m/z* bins. Correction methods (1), (2), and (3) are obtained through Equations 7, 9, and 12, respectively, of the Supplementary Information.

## Results and Discussion

We can evaluate these methods of TDC saturation correction by assessing how well their corrected values adhere to those that would be expected from the theoretical isotope patterns of a known compound. More direct methods of evaluation are difficult for real data since the true ion count is unknown. We will only illustrate correction methods (2) and (3) on real data, as the shape of the chromatographic peaks is not understood adequately that we can use method (1) reliably. The mass peak will be modeled as Gaussian although this functional form is only an approximation of the true shape at high ion counts.

When working with LC/MS data, the performances of methods (2) and (3) can be evaluated by applying them to the mass peaks of a single compound that induces a very strong signal. This is because methods (2) and (3) operate on the mass peak observed over individual chromatographic scans, and for strong signals these mass peaks will typically range from very small, near the edges of the chromatographic peak, to very large, near its zenith. Therefore, the accuracy of the correction methods can be examined over the full range of intensities that are likely to be encountered under standard experimental settings with such data. Since it is primarily the intensities of the mass peaks rather than the identities of the compounds inducing them that affect the performance of the corrections, applying the correction methods to additional compounds would yield little additional information. The signal induced by salicylic acid was identified as being amongst the strongest present in an LC/TOFMS data set derived from a sample of synthetic urine as part of an experiment described elsewhere [8]. Therefore, the two lowest-mass isotopologues of salicylic acid (shown on the heatmap in the Supplementary Information Figure 1) were used to validate methods (2) and (3).

**Figure 1:**
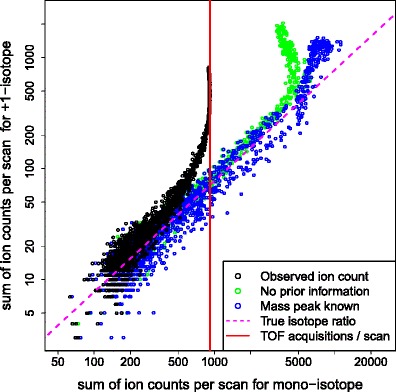
Scatterplot obtained by plotting the raw (black) ion counts of the two isotopologues of salicylic acid against each other, along with the intensities estimated via correction methods (2) and (3) (blue and green, respectively). The magenta line indicates the true isotope ratio to which all of these should conform, whereas the red line indicates the number of TOF acquisitions for each chromatographic scan

The sum of ion counts observed across the mass peaks of each of the two isotopologues are plotted against each other for matching chromatographic scans on Figure 1 along with the theoretical isotope ratio for salicylic acid. The raw ion counts adhere to the predicted isotope ratio for low intensities, but deviations become increasingly severe as the ion count of the monoisotope approaches the number of TOF acquisitions per chromatographic scan, which corresponds to full saturation. The estimated intensities provided by correction methods (2) and (3) are also shown. For low ion counts, these are only marginally greater than the raw ion counts; however, they largely restore the correct isotope ratio for monoisotopic intensities of up to around 4000 for correction method (3) and 5000 for correction method (2). Although there are substantial deviations from the true isotope ratio at the most heavily saturated scans, these two correction methods clearly provide a strong improvement over the isotope ratio suggested by the raw ion counts and, thereby, provide further support for the validity of the basic model.

The discrepancies from the true isotope ratios at high intensities are due to the heavy non-Gaussian tails of the largest mass peaks, which exceed the duration of the detector dead time as is explained in the experimental section of the Supplementary Information. However, for instruments for which the dead time exceeds the mass peak width and for which the mass and chromatographic peak shapes conform to known mathematical functions, a much better effective dynamic range may be attained by the very same corrections. Although we are only able to illustrate this with simulated data, they are derived from the basic model, which we believe provides the closest approximation to the true distribution of raw TOFMS data that has been published to date. Moreover, we know what its limitations are and it is likely possible to address them with engineering solutions. The following simulations, therefore, demonstrate potential improvements that may be within reach if instruments are devised whose data can be modeled more accurately.

**Figure 2:**
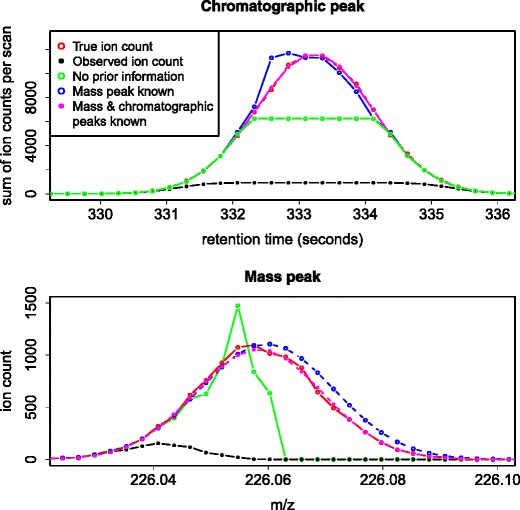
True (red) and observed (black) ion counts generated via the basic model, along with the statistical corrections obtained when both the functional forms of the mass and chromatographic peaks are known (magenta), when only the mass peak is known (blue) and when no such prior information is available (green). The plot on top shows the peak in the chromatographic dimension with ion counts in the *m/z* dimension having been summed. The plot on the bottom shows the mass peak observed in a single chromatographic scan

The plots on Figure 2 show the true and the observed ion counts of a simulated peak in the chromatographic and *m/z* dimensions, along with the results of all three correction methods. Despite the very heavy saturation, correction methods (1) and (2) provide good estimates of the true rate of ion arrivals. It is to be expected that correction method (1) would perform best as it can reliably synthesize observations from multiple chromatographic scans with knowledge of the general variation in the chromatographic dimension. Correction method (3) performs well at the low-mass sides of mass peaks, where a large number of the TOF acquisitions are capable of registering ions, but poorly at the high-mass ends, where most of these are unavailable due to dead time.

The more general performances of correction methods (2) and (3) depend on numerous factors, including the TDC time resolution, the width of the mass peaks, and whether or not the latter quantity is known in advance or must be estimated from the data. Correction method (2) generally provides a modest improvement over method (3). For very high intensities, the latter will reach a plateau (see Figure 2), whereas the former will exhibit very high variance. However, correction method (1) provides highly accurate estimates for all realistic settings that we have examined. Even for peaks of 10^7^ ions (100 times larger than the one shown on Figure 2), an excellent fit is obtained, amounting to an enhancement in effective dynamic range of over four orders of magnitude. Such improvements may be compared with those achieved via (potentially costly) engineering solutions, which in [9] enhance the detection efficiency by a factor of around 2.5 and in [10] increase the dynamic range by about one order of magnitude. We therefore believe that a strong case can be made for devoting further efforts to addressing the problem of TDC saturation via statistical corrections.

## Conclusions

Our results suggest that if mass and chromatographic peaks were sufficiently well characterized that correction method (1) could be properly applied to real data, the constraints on TDC dynamic range, which are currently severe, would be very greatly reduced. Given that TDCs generally have better time resolution and are cheaper than the alternative ADCs, this would be an important development. However, to realize it more extensive efforts must be made at understanding the full intricacies of the data generation process, particularly of the ion optics and of the chromatography, so that the peak shapes can be more thoroughly characterized. We hope that our theoretical results will help to motivate greater emphasis on the development of a more complete mathematical description of mass spectrometry data.

## Electronic supplementary material

Below is the link to the electronic supplementary material.

ESM 1 (DOCX 972 kb)
