# Supplementary material for: Orders of Magnitude Extension of the Effective Dynamic Range of TDC-Based TOFMS Data Through Maximum Likelihood Estimation
Source: J Am Soc Mass Spectrom. 2014 Jul 22;25(10):1824–7. doi: 10.1007/s13361-014-0961-5 (PMC4161928; doi:10.1007/s13361-014-0961-5)
Supplement: Supplementary file 1 — (DOCX 972 kb) [file 13361_2014_961_MOESM1_ESM.docx]

**Theory**

We provide a brief summary of the basic model whose detailed derivation is given in [1]. Let *k_i,j_* denote the recorded ion count at the *i*th chromatographic scan and *j*th tick of the TDC clock (which measures the time-of-flight) with reference to the first scan and first tick at which a compound is observed in the data. *k_i,j_* is obtained by summing all the ions observed in *V_i,j_* distinct TOFMS data acquisitions. Only a value of either 0 or 1 can be recorded in each TDC tick of each acquisition, regardless of how many ions actually arrive in that time interval. Therefore *k_i,j_* has a Binomial distribution where the sample size is *V_i,j_* and the Binomial probability, *ρ_i,j_*, is the probability of “one or more” ion arrivals:

$k_{i,j}\sim\left(\begin{matrix} V_{i,j} \\ k_{i,j} \end{matrix}\right)\rho_{i,j}^{k_{i,j}}\left(1-\rho_{i,j}\right)^{V_{i,j}-k_{i,j}}$ **(1)**

Following each ion arrival, TDC-based detectors suffer from a period of dead time during which they are unable to record further ions. Therefore the sample size, *V_i,j_*, which tells us how many TOFMS data acquisitions are capable of recording ion arrivals at the *i*th scan and *j*th tick, will decrease over the course of a mass peak. In the first tick of a given mass peak it will equal the total number of TOFMS acquisitions that are histogrammed to form a single spectrum, which we label *N_p_*. But in the ensuing ticks we must subtract the sum of all preceding ion counts since a corresponding number of TOFMS acquisitions will be unable to record additional ions due to dead time. This can be written

$V_{i,j}=N_{p}-\sum_{x=1}^{x=j-1} k_{i,x}$ **(2)**

where we assume that the dead time exceeds the width of the mass peak. Since the rate of ion arrivals is governed by the Poisson distribution, the Binomial probability, *ρ_i,j_*, of one or more ion arrivals in a given tick is given by

$\rho_{i,j}=1-P\left(\text{zero ion arrivals}\right)=1-\frac{\left(\Lambda_{i,j}/N_{p}\right)^{0}e^{-\Lambda_{i,j}/N_{p}}}{0!}=1-e^{-\Lambda_{i,j}/N_{p}}$ **(3)**

where Λ*_i,j_* is the discretized Poisson rate function, which describes the expected number of ion arrivals over the *N_p_* acquisitions that are histogrammed to constitute the *i*th scan and the *j*th tick. This can be written

$\Lambda_{i,j}=I\int_{\tau_{i}}^{\tau_{i}+\tau_{\varepsilon}} \Gamma\left(\tau,\theta_{\Gamma}\right)d\tau\int_{t_{j}}^{t_{j+1}} \Omega\left(t,\theta_{\Omega}\right)dt$ **(4)**

where *I* is the expected number of ion arrivals over the entire peak. Ω(*t, θ*_Ω_) and Γ(*τ, θ*_Γ_) are normalized functions describing the shapes of the mass and chromatographic peaks respectively. *t* denotes the time-of-flight and *τ* the retention time. The *t_j_* and *τ_i_* enumerate the ticks of the TDC clock and the chromatographic scans respectively and *τ_ε_* is the chromatographic scan time. The location, scale and other parameters that determine the precise shapes of these functional forms are given by the vectors *θ*_Ω_ and *θ*_Γ_. It is likely that Ω and Γ exhibit some dependence on *I*, when the latter is large.

If we observe the ion counts *k_1,1_*,…, *k_N,M_* across a peak in both the retention time and time-of-flight dimensions, we can simply write the probability distribution of the entire peak as the product

$P\left(k_{1,1},...,k_{N,M}|\theta_{\Omega},\theta_{\Gamma},I\right)=\prod_{i=1}^{N} \prod_{j=1}^{M} \left(\begin{matrix} V_{i,j} \\ k_{i,j} \end{matrix}\right)\rho_{i,j}^{k_{i,j}}\left(1-\rho_{i,j}\right)^{V_{i,j}-k_{i,j}}$ **(5)**

More elaborate features of LC/MS data can easily be modeled by expanding the above equations appropriately. For example, the distinct peaks observed in isotope and fragmentation patterns are characterized by having essentially the same elution profiles and this can be expressed by using identical values for the *θ*_Γ_‘s of these peaks. Similarly, an isotopic abundance pattern may be described by fixing the ratios between the *I*’s of the peaks to correspond to this pattern [2,3].

We have not provided specific mathematical expressions for the mass and chromatographic peaks in equation 4, because these depend on the instruments used and their functional forms do not appear to be known to a very high degree of accuracy although some proposals have been made [4–6]. For the instrument used in this study, we demonstrated in [1] that a Gaussian function can provide a good approximation to the mass peaks for moderate ion counts, but for higher intensities their tails were found to be heavier than Gaussian. The basic model can be used to correct for TDC saturation without precise knowledge of the peak shapes, Ω and Γ, but the attainable correction is far better if they are known – to the point where it could conceivably make TDC-based TOF instruments significantly more competitive with their ADC-based counterparts. We cannot comment on how extensively the functional forms of Ω and Γ have been studied in industry, but we do hope to demonstrate with this communication that finding accurate expressions for them may be a much more important task than has generally been assumed in academia, judging from the relatively limited efforts devoted to the problem. In the context of TDC saturation correction it is especially important to understand the shape of Ω when the ion count is very high, and this will require careful analysis of the ion optics which will be further complicated when space charge effects become substantial.

It is not uncommon for MS data models to be validated through rather informal procedures, such as plotting them alongside empirical data and arguing that the two “look alike”. In contrast, the basic model has been validated through what we believe may be one of the most demanding validation procedures to which any model of MS data has been subjected. Since the basic model is derived from first principles it aims to capture the full distribution of the empirical data and thereby enable the proper use of key tools of statistical theory, including hypothesis testing. As we demonstrated in [1] through a thorough analysis of the empirical distributions of the test statistic used and of their associated p-values, the basic model can be used reliably in its current form to perform formal hypothesis testing of LC/TOFMS data-analytical problems for moderate ion counts. This would not be possible if the model did not provide a very close approximation of the true distribution of the data analyzed.

*Three methods of TDC saturation correction*

An important potential application of the basic model is the estimation of the true rate of ion arrivals over each tick (Λ*_i,j_*) from the observed ion count (*k_i,j_*) when there is substantial TDC saturation. In some cases the expected number of ion arrivals over the entire peak, *I*, may be of more direct interest than the Λ*_i,j_* and this quantity can easily be estimated as well. TDC-based systems are frequently criticized for their limited dynamic range and a number of elaborate engineering solutions have been devised to accommodate for it [7–10]. But the need for such workarounds might be significantly reduced if further efforts were instead made at devising accurate probability distributions for the data so that algorithms such as those described below can be used to better extract the information they contain.

We consider three corrections all of which are MLEs, but corresponding to the scenarios in which 1) the shapes of both the mass and chromatographic peaks are known, 2) the mass peak is known but the chromatographic peak is not, and 3) neither shape is known. Many further variations may be conceived depending on the specific sets of parameters that are known, and on the degree to which their relations are understood. In addition, experimental features that are in principle predictable might eventually be used to impose constraints on the values of the parameters. For example, if the degree of broadening of mass peaks with increasing m/z, or of chromatographic peaks with increasing elution time, could be specified mathematically, a smaller subspace of *θ*_Ω_ or *θ*_Γ_ could be searched in the maximization of the likelihood, since there would be a direct relationship between the location and scale parameters for Ω or Γ. This would allow for more accurate estimates and would reduce computational demands. Specifying additional features, such as the change in mass peak width with increasing charge state would provide similar benefits.

The application of these correction methods might also be tailored to exploit prior information arising from specific analytical tasks. For example, in the case of LC/MS/MS, where all the fragments will share the elution profile of the precursor ion (and therefore also share the *θ*_Γ_ of the precursor) correction method (1) might be applied to the observed ion counts of all the different fragments simultaneously. Distinct sets of *θ*_Ω_ parameters must still be estimated for each fragment since these have different masses, but only one set of *θ*_Γ_ parameters need to be estimated, which means that there are effectively more (and more diverse) data available for the estimation of each parameter, which can lead to significantly more accurate estimates. Similar points apply to signals derived from different isotopologues of the same compound. The basic model may also be used to determine which signals are likely to be derived from the same precursor in the first place, by determining whether their elution profiles could be identical, through a formal test of hypothesis, as is discussed in detail in [1].

However, in the present study we choose to focus on the three simpler correction methods mentioned above because they constitute a natural and more general set of distinct correction standards, the best being method (1), whose use will require substantial improvements in the understanding of our instruments and the poorest being (3) which is immediately available to us. These methods can be used to correct the intensities of signals due to any compound, including product ions in LC/MS/MS experiments and it should be noted that they can provide corrections for m/z bias as well as intensity bias.

*1) Mass and chromatographic peak shapes known*

The greatest improvement in effective dynamic range that the basic model can provide is obtained when both Ω and Γ are known, since this allows us to incorporate extensive prior knowledge into the estimates of the Λ*_i,j_*. In this scenario we simply rewrite equation 5 as a likelihood function:

$L\left(\theta_{\Omega},\theta_{\Gamma},I\left|k_{1,1},...,k_{N,M}\right.\right)=\prod_{i=1}^{N} \prod_{j=1}^{M} \left(\begin{matrix} V_{i,j} \\ k_{i,j} \end{matrix}\right)\rho_{i,j}^{k_{i,j}}\left(1-\rho_{i,j}\right)^{V_{i,j}-k_{i,j}}$ **(6)**

and, numerically, find the values of *θ*_Ω_, *θ*_Γ_ and *I* that maximize it, given the observed *k_1,1_*,…, *k_N,M_*. From this, MLEs of the Λ*_i,j_* can be obtained by replacing *θ*_Ω_, *θ*_Γ_ and *I* by their estimates in equation 4:

$\hat{\Lambda}_{i,j}=\hat{I}\int_{\tau_{i}}^{\tau_{i}+\tau_{\varepsilon}} \Gamma\left(\tau,\hat{\theta}_{\Gamma}\right)d\tau\int_{t_{j}}^{t_{j+1}} \Omega\left(t,\hat{\theta}_{\Omega}\right)dt$ **(7)**

*2) Mass peak shape known, chromatographic peak shape unknown*

In the scenario where Ω is known, but Γ is not, we lack prior knowledge of the variation in the expected ion count as a function of the retention time. But for a single mass peak at the *i*th scan, the likelihood can be written

$L\left(\theta_{\Omega,i},I_{i}\left|k_{i,1},k_{i,2},...,k_{i,M}\right.\right)=\prod_{j=1}^{M} \left(\begin{matrix} V_{i,j} \\ k_{i,j} \end{matrix}\right)\rho_{i,j}^{k_{i,j}}\left(1-\rho_{i,j}\right)^{V_{i,j}-k_{i,j}}$ **(8)**

with

$\Lambda_{i,j}=I_{i}\int_{t_{j}}^{t_{j+1}} \Omega\left(t,\theta_{\Omega,i}\right)dt$ **(9)**

where *I_i_* denotes the average number of ion arrivals of the compound in question over the entire *i*th scan. We can then obtain MLEs of *θ*_Ω,_*_i_* and *I_i_* and use them to estimate Λ*_i,j_,* as above.

*3) neither peak shape known*

Maximizing the likelihood turns out to be particularly simple if neither Ω nor Γ are known, as it is then possible to obtain an analytical expression for the supremum of the likelihood function. In fact, for systems for which the expression for *V_i,j_* given by equation 2 applies, it turns out that this MLE is very similar to a dead time correction algorithm proposed by Coates [11,12]. However, Coates’ derivation was based on heuristic arguments rather than statistical theory, and his correction is not generally an MLE. We note that another correction algorithm bearing close resemblance to that of Coates and to this MLE was later proposed by Stephan et al. [13] and was recently more explicitly formulated as a Binomial-based method by Keenan et al. [14].

According to the basic model, each observed ion count *k_i,j_* is the outcome of a Binomial distribution with sample size *V_i,j_* and if Ω and Γ are unknown, each Binomial probability, *ρ_i,j_*, must be estimated independently. The MLE is

$\hat{\rho}_{i,j}=\frac{k_{i,j}}{V_{i,j}}$ **(10)**

and since

$\rho_{i,j}=1-e^{-\Lambda_{i,j}/N_{p}}$ **(11)**

we have

$\begin{matrix} \hat{\Lambda}_{i,j}=-N_{p}\log\left(1-\hat{\rho}_{i,j}\right) \\ \\ =-N_{p}\log\left(1-\frac{k_{i,j}}{V_{i,j}}\right) \end{matrix}$ **(12)**

which is essentially equivalent to the correction proposed in [11] and generalized in [12], if the detector dead time period always exceeds the combined duration of the TDC ticks investigated. A problem immediately arises with this algorithm if ions are registered by all of the *N_p_* acquisitions over the course of a mass peak. In this scenario we must have *k_i,j_* = *V_i,j_* at the tick where the last ions are registered, which yields an infinite “corrected” value of Λ*_i,j_* and meaningless estimates at all ensuing ticks. Although this can be avoided (e.g. by replacing *N_p_* by *N_p_*+1 in equation 2), it is a deficiency that highlights the importance of incorporating prior knowledge of the data into the estimators used.

**Experimental Details**

The experiment was run in triplicate on a Waters Q-TOF Premier, and the mass peaks of salicylic acid observed across 460 distinct chromatographic scans of each run were used. For the instrument used in this experiment the TDC time resolution is 278 picoseconds, and the dead time lasts about 5 nanoseconds (i.e. about 18 TDC ticks). The total number of TOF acquisitions per chromatographic scan, *N_p_* was 915. A heatmap of the data is shown on Figure 1, along with a representative, mildly saturated mass peak.


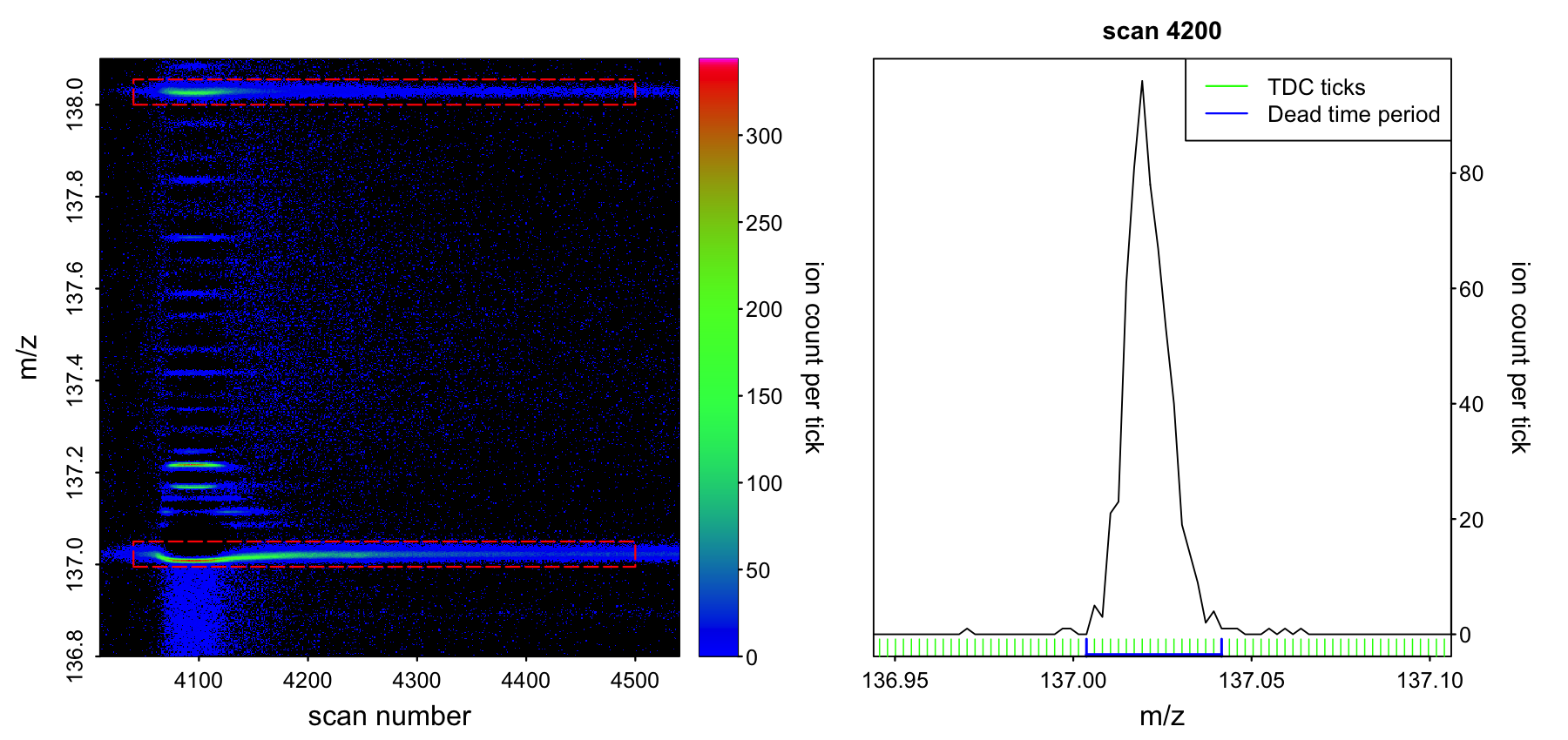


Figure 1 – Left: heatmap of the raw ion counts observed for the two lowest-mass isotopologues of salicylic acid. The heavy tails of the mass peaks are evidenced by the large number of ions detected far from the modes of the mass peaks. The regions contained in the red rectangles indicate the *k_i,j_* used to assess the isotope accuracy for both the raw data and the corrections, and it can be seen that these include mass peaks of a very wide range of intensities. Right: monoisotopic mass peak for the 4200^th^ chromatographic scan, with the TDC ticks and typical dead time duration indicated.

The mass peaks due to the two isotopologues are visible over a wide range of chromatographic scans near m/z = 137 and m/z = 138. The severe saturation near the chromatographic zenith of the monoisotopic peak is evident from the complete absence of detected ions, at m/zs just above it, which suggests that all TOFMS acquisitions are saturated at that point. The weaker signals that can be observed at higher m/zs are artifacts that are due to detector “ringing“ resulting from the very strong detector response to the monoisotope. Since this phenomenon occurs after the m/z range on which the correction method operates it does not distort it.

However, the heavy tails of the largest mass peaks, which are evidenced by the large number of ions detected near the chromatographic zenith, but at masses substantially lower than 137, make it difficult to determine *V_i,j_*. This is because some of the acquisitions that were blocked due to dead time are able to reopen over the course of a single mass peak, in violation of equation 2. For the purposes of this study the *V_i,j_* were therefore estimated by subtracting from *N_p_* the ion counts just prior to the *k_i,j_*, over a period matching the dead time of the instrument. Although a more elaborate expression for *V_i,j_* than equation 2 can be derived [15], a more promising approach might be to increase the duration of the dead time and to reduce the tails of the mass peaks so that the probability of the dead time period ending over the course of one is negligible. Those are solutions that must be provided through engineering efforts.

**Simulation Details**

The mass peak was modeled as Gaussian and the chromatographic peak was modeled as skew-normal [16], the latter function having greater computational stability than other models, such as the exponentially modified Gaussian [17], which is important when maximizing the likelihood. The peak parameters were derived by fitting a Gaussian and a skew-normal function to a relatively unsaturated mass peak of the +1-isotopologue of nitrotyrosine, observed in the experimental data set described above. This resulted in the parameter sets Ω, = (226.0541, 0.011) (location, scale, where the dimension is m/z), Γ = (333, 0.91, 0.34) (location, scale, shape, where the dimension is seconds). The total number of acquisitions per chromatographic scan, *N_p_* was set to 915 and the TDC time resolution was set to 278 picoseconds, matching the settings of the instrument used. However the total expected ion count was set to 10^5^ (about 20 times the ion count of the observed nitrotyrosine peak), resulting in a simulated peak that exhibits very heavy saturation. For correction method (3), *N_p_* was set to 916 in equation 2 in order to avoid the infinities that would inevitably be obtained otherwise. For the two other methods, the resulting likelihood functions were maximized using a Newton-based method.

**References**

1. Ipsen, A., Ebbels, T.M.D.: Prospects for a Statistical Theory of LC/TOFMS Data. J. Am. Soc. Mass Spectrom. **23**(5), 779–791 (2012)

2. Ipsen, A., Want, E.J., Lindon, J.C., Ebbels, T.M.D.: A Statistically Rigorous Test for the Identification of Parent−Fragment Pairs in LC-MS Datasets. Anal. Chem. **82**(5), 1766–1778 (2010)

3. Ipsen, A., Want, E.J., Ebbels, T.M.D.: Construction of Confidence Regions for Isotopic Abundance Patterns in LC/MS Data Sets for Rigorous Determination of Molecular Formulas. Anal. Chem. **82**(17), 7319–7328 (2010)

4. Li, J.: Comparison of the Capability of Peak Functions in Describing Real Chromatographic Peaks. J. Chromatogr. A **952**(1–2), 63–70 (2002)

5. Opsal, R.B., Owens, K.G., Reilly, J.P.: Resolution in the Linear Time-of-Flight Mass Spectrometer. Anal. Chem. **57**(9), 1884–1889 (1985)

6. Strittmatter, E.F., Rodriguez, N., Smith, R.D.: High Mass Measurement Accuracy Determination for Proteomics Using Multivariate Regression Fitting:  Application to Electrospray Ionization Time-Of-Flight Mass Spectrometry. Anal. Chem. **75**(3), 460–468 (2003)

7. Barbacci, D.C., Russell, D.H., Schultz, J.A., Holocek, J., Ulrich, S., Burton, W., Van Stipdonk, M.: Multi-Anode Detection in Electrospray Ionization Time-of-Flight Mass Spectrometry. J. Am. Soc. Mass Spectrom. **9**(12), 1328–1333 (1998)

8. Green, M., Jackson, M.: Mass Spectrometer and Methods of Mass Spectrometry. US6894275 B2, (2005)

9. He, Y., Poehlman, J.F., Alexander, A.W., Boraas, K., Reilly, J.P.: One Hundred Anode Microchannel Plate Ion Detector. Rev. Sci. Instrum. **82**(8), 085106 (2011)

10. Loboda, A.: Method and System for Operating a Time of Flight Mass Spectrometer Detection System. WO2011095863 A3, (2011)

11. Coates, P.B.: The Correction for Photon `pile-up’ in the Measurement of Radiative Lifetimes. J. Phys. [E] **1**(8), 878 (1968)

12. Coates, P.B.: Analytical Corrections for Dead Time Effects in the Measurement of Time-interval Distributions. Rev. Sci. Instrum. **63**(3), 2084–2088 (1992)

13. Stephan, T., Zehnpfenning, J., Benninghoven, A.: Correction of Dead Time Effects in Time-of-flight Mass Spectrometry. J. Vac. Sci. Technol. A **12**(2), 405–410 (1994)

14. Keenan, M.R., Smentkowski, V.S., Ohlhausen, J.A. (Tony), Kotula, P.G.: Mitigating Dead-Time Effects during Multivariate Analysis of ToF-SIMS Spectral Images. Surf. Interface Anal. **40**(2), 97–106 (2008)

15. Ipsen, A.: Probabilistic Modelling of Liquid Chromatography Time-of-Flight Mass Spectrometry. Ph.D., Imperial College London, (2011)

16. Azzalini, A.: A Class of Distributions Which Includes the Normal Ones. Scand. J. Stat. **12**(2), 171–178 (1985)

17. Lan, K., Jorgenson, J.W.: A Hybrid of Exponential and Gaussian Functions as a Simple Model of Asymmetric Chromatographic Peaks. J. Chromatogr. A **915**(1–2), 1–13 (2001)
